# One-year discontinuation among users of the contraceptive pill and injection in South East Tigray region, Ethiopia

**DOI:** 10.1371/journal.pone.0285085

**Published:** 2023-05-01

**Authors:** Kahsay Zenebe Gebrslasie, Gebretsadik Berhe, Haftom Taddese, Solomon Weldemariam, Gelawdiyos Gebre, Abdella Amano, Daniel Birhane, Gebrehud Berihu, Munir Kassa, Mulu Weldegebriel, Ezana Haile

**Affiliations:** 1 Mekelle University College of Health Sciences, Mek’ele, Ethiopia; 2 College of Medicine and Health Sciences, School of Public Health, Hawassa University, Hawassa, Ethiopia; 3 Federal Ministry of Health, Addis Ababa, Ethiopia; 4 Institution of Engineering and Technology Michael Faraday House, Stevenage, United Kingdom; Makerere University School of Public Health, UGANDA

## Abstract

**Objectives:**

The aim of this study is to determine the 12 months’ discontinuation rate and associated factors among family planning clients using pills and injection.

**Methods:**

A follow-up study was initiated to collect data from 845 family planning users between November 2017 and December 2018. An interviewer administered questionnaire was used to collect data from participants. Data were entered into EpiData version 3.1 and analyzed using SPSS version 20, where both are open-source systems. A Cox proportional-hazards model was used to estimate the hazard ratios (HR) for the rate of discontinuation among participants.

**Result:**

At 12 months, 63.5% of women discontinued the use of their baseline method. For the individual methods, 84% of women that chose the pill discontinued its use and for those using the injectable, 60.7% of women discontinued its use. Using the adjusted Cox proportional-hazards model, pills users (HR = 1.77; 95%CI = [1.4–2.3]), users receiving family planning services in the same room as other maternal health clinic services (HR 1.58; 95%CI = [1.16–2.2]), users served by health officers (HR = 3.7; 95%CI = [1.66–8.2]), and users not intending to use the baseline method continuously (HR = 1.6; 95%CI = [1.16–2.24]) were significantly more likely to discontinue using the baseline method. The main reason cited for discontinuation was side effects of contraception.

**Conclusions:**

The discontinuation rate of the baseline contraceptive method after 12 months was very high. To increase the continuity of contraceptive use, family planning services should be given in a separate room with effective counseling on potential side effects, provided by midwives or nurses who have good counseling skills.

## Introduction

The United Nations’ median projection estimated that the world population will reach 8.5 billion by 2030 and Africa has the highest population size projection [1]. Ethiopia is one of the countries with high population growth. Ethiopia adopted a population policy in 1993 for population growth management. The aim of this policy was to find the balance between population growth and socioeconomic development [2].

Ethiopia’s national population policy plan was to reduce fertility rate from 7.7 children per woman in 1990 to 4.0 children per woman in 2015 and to increase contraceptive prevalence from 4% in 1990 to 44% in 2015 [2]. According to the 2019 mini Ethiopian Demographic and Health survey (EDHS) the Contraceptive Prevalence Rate (CPR) was 41% in Ethiopia and 37.3% in Tigray Region [3].

Contraceptive discontinuation is defined as “the termination of episodes of use of any reversible contraceptive method which could end up with abandoning use, failure or switch to other method [4]. In less developed countries, around 214 million women are not using contraception, mainly due to discontinuation, i.e. discontinuing using a contraceptive method within 12 months of commencing a specific episode of use. This also apparent in situations where pregnancy is not intended [4]. Early discontinuation has been the main factor for the low CPR [5] that leads to a high rate of unintended pregnancies [6]. Analysis from the Demographic and Health Surveys (DHS) revealed that in women with unmet needs for modern family planning, 38% of these were due to discontinuation of contraceptive use [7]. Similarly, according to the 2016/2011 EDHS, the contraceptive discontinuation rate was 37% within 12 months. This study focused on pills and injections because it has the highest discontinuation rate in Ethiopia, i.e. the discontinuation rate for pills was 70% and for injections 34.2%, all contributing to low CPR [2]. The prevalence of use of injections and of pills user were 27% and 2%, respectively.

Several studies revealed that age, occupation, educational status, marital status, quality of service and side effects were significantly associated with the discontinuation rate of contraception [5, 8–10]. Notably, there has been no other study on the prospective follow-up of contraception use in Ethiopia.

Understanding the factors associated with discontinuation rate of contraceptive pills and injections can be used to make improvements in the delivery of family planning services, and hence increase the use of contraceptive methods. Thus, the aim of this study was to assess the 12-month discontinuation rate of pills and injections, together with the associated factors among users from South East Tigray Region, Ethiopia.

## Materials and methods

### Definitions

**Method continuation** refers to continuous use of the baseline method at each survey interval without a period of discontinuation for more than one month.

**Method discontinuation** refers to no longer using the baseline method at any of the follow-up surveys, a temporary discontinuation of the method for at least one month or switching to another method.

### Study area and period

The study was conducted from November to December 2018 among the users of contraceptive pills and injections in the South East Zone of Tigray Region. Tigray Region is located 783 km from north of Addis Ababa, the capital city of Ethiopia. Based on the 2007 census, the total population was estimated to be 4,316,988. Women of childbearing age (15–49) comprise 251,650 of the population. According to the 2015 Tigray Regional Health Bureau annual report, the healthcare service consists of; one specialized hospital, 15 general hospitals, 22 primary hospitals, 204 health centers, and 712 health posts. In addition, there are three private hospitals. In South East Tigray there are 25 health centers, 104 health posts and one hospital. The contraceptive prevalence rate of the region was 37.3% as reported in the 2019 mini EDHS [3].

### Study design

An institution-based prospective follow-up study was conducted.

### Study population

All participants in the study were users of contraceptive pills or injections, which had been dispensed from governmental health facilities in the South East Zone of Tigray, and who had no desire to conceive within 12 months of their recruitment to the study, while being sexually active with a partner.

### Sample size determination and sampling technique

The required sample size for this study was determined using the single population formula in the open source software, Epi Data Info version 3.03. The assumptions used to calculate the sample size were a continuation rate of 49% [11], 95% confidence interval, a 5% margin of error, a design effect size of 2 and a 10% expected loss to follow-up.


$$\text{n}=\frac{\left(z\right)2p\left(1-p\right)}{d2}=384\text{x}2\;\left(\text{design}\;\text{effect}\right)=768\;\text{plus}\;10\;\%;\;\text{the}\;\text{final}\;\text{sample}\;\text{size}\;\text{was}\;845.$$

Health facilities were stratified into the settings of hospital, health center and health post. A simple random sampling technique was used to select 1 hospital (Adigudom hospital), 4 health centers (Adikeyh, Hiwane, Romanat, Samre) and 15 health posts (Arabay, Simret, Melsa, Selam, Felegeselam, Lemlem, Meseret, Romanat, Samre, Freweyane, Waza, Gijet, Bamba, Dekera, Addissalem) from an available pool of 1 hospital, 25 health centers and 104 health posts. The average client load (2535) for each health facility was taken for the 2 months preceding recruitment, and a recruitment target was proportionally allocated to each health facility based on this client load and in order to find the weighting. Eligible participants from each health facility were selected using systematic sampling of every 3^rd^ pill and injection user until the calculated sample of 845 participants was reached. For the purpose of follow-up, selected users were requested to give their contact address (participant / representative phone number, and also the name and phone number of their assigned health extension worker). The follow-up was performed every four months (i.e. at 4, 8 and 12 months).

### Schematic presentation of sampling procedure (Fig 1)

**Fig 1:**
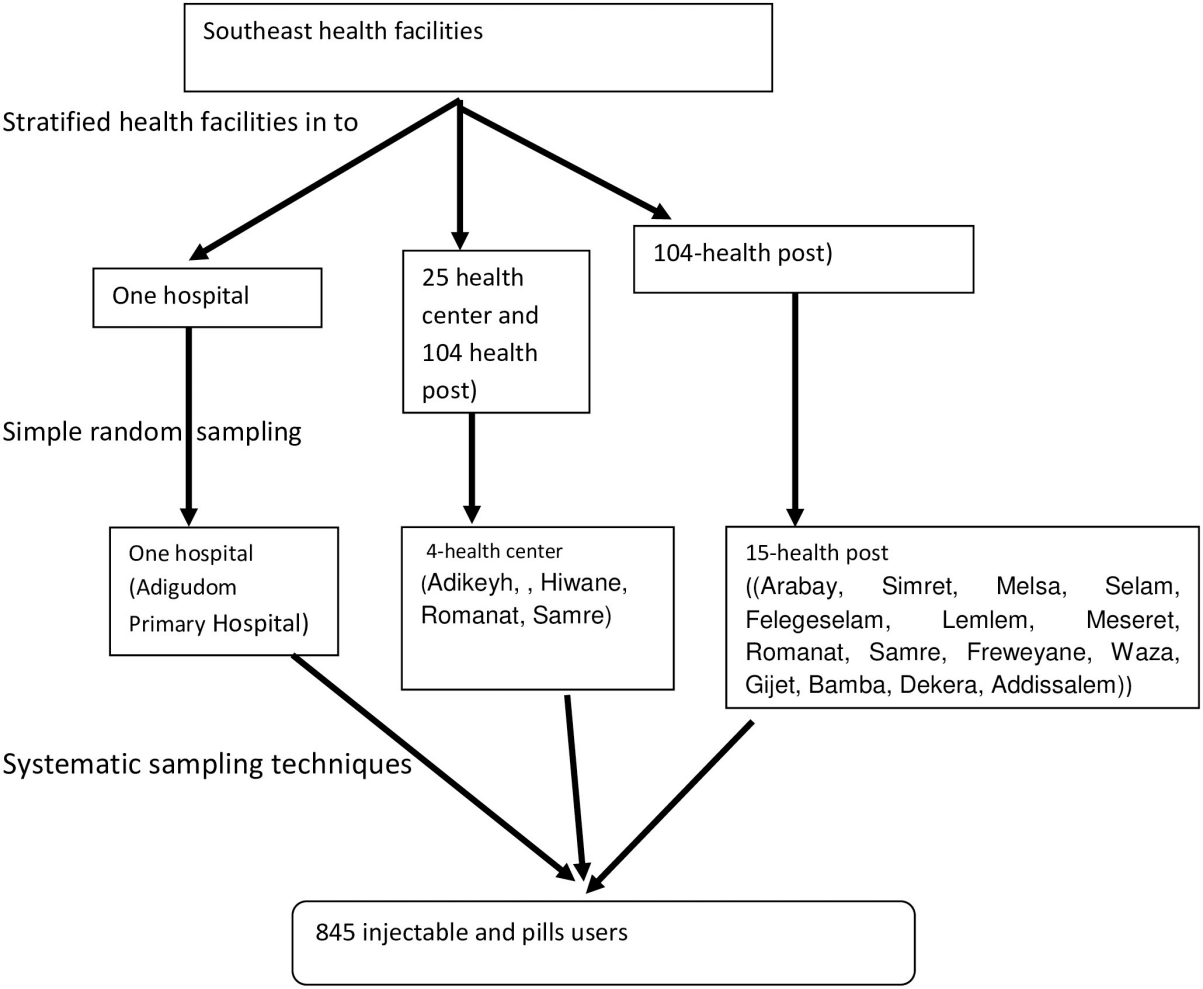
Shows that sampling procedures to select study participants.

### Data collection and processes

The questionnaire was developed after reviewing different researches and literature. A questionnaire was prepared in English, translated to Tigrigna and translated back to English by the same language expert to ensure that consistency of the questions had been maintained.

Data were collected through the administration of a structured interview. The interview was made in a private place to avoid fear or discomfort. Pretest was done on 10% of the total sample size to know if there is confusion on items or problems for improvement of the data collection tool. Training was given for both data collectors and supervisors about the aim of the study, procedures, and how to approach the study participants and data collection techniques. For validation purposes the questionnaire was evaluated by experts for its clarity and ease of data collection and whether it covered all aspects of the study.

Baseline data were collected by 20 midwives holding diploma-level qualifications, and supervised by 5 midwives holding degree-level qualifications. A five-day training course was given for data collectors and supervisors. After collecting baseline information, participants completed the structured interview at 4, 8 and 12 months.

#### Measurement

The questionnaire covered socio-demographic factors, factors relating to the family planning service, the participant’s obstetrics and gynecological history, the accessibility of the family planning service, and factors associated with the method of contraception used. The data collectors assessed the health facilities for the availability of separate rooms for family planning, the professional role (health extension worker / midwife / nurse / health officer) and qualifications (Diploma / BSc / Master) of the provider, and their level of experience working in that role.

The follow-up comprised; whether the participant had continued to use that method of contraception, their level of satisfaction with the method, the reason for discontinuation where applicable and the identification of a different method in the case of switching. These factors were assessed at each follow-up by telephone survey or in-person interview at the health center.

### Data management and analysis

Questionnaire responses were checked for completeness and for clarity inconsistency, missing entry and repeated entry. Data were entered to the open-source software EpiData version 3.1 then coded and cleaned. The data were exported to the open-source system SPSS version 20 for analysis. Descriptive statistics were summarized in tabulated format. The Kaplan-Meier curve was used to compare the continuation rate of pills versus injections.

Unadjusted and adjusted Cox proportional-hazards a ratio was used to assess factors associated with discontinuation of contraception use after one year. The proportional-hazards model was updated to include; participant age (in years), type of contraception used (pills / injections), the availability of a separate health facilities room for family planning (yes / no), profession of the family planning provider (health extension worker / midwife / nurse / health officer), the intention to use the baseline method continuously (yes / no), whether information was provided on when their next appointment was, and how long they had previously been using the baseline method for. Participants lost to follow-up were considered as censored at their last completed survey date, and their data up until the point of last contact were included in the study. Statistical significance was p<0.05 with a 95% CI.

### Ethical considerations

Ethical approval was obtained from the institutional review board (IRB) of Mekelle University, College of Health Sciences. Additionally, a letter of permission was taken to each participating health facility in South East Tigray. Verbal consent was obtained from each study participant prior to commencing the data collection process. The consent forms included the purpose of the study, the procedure, risk or any discomfort and benefit one gets out of the study. Any participants not willing to take part in the study were not coerced. They were also informed that all data obtained from them would be kept confidential and was only being gathered for the purpose of the study. The consent form was given approval by MUCHS Ethics committee.

## Results

Among the 845 recruited participants, 831 women were interviewed and enrolled, with a response rate of 98.3%. The number of women interviewed at 4, 8 and 12 months was 828 (99.6%), 630 (76.0%) and 429 (51.9%) respectively. The age range for the majority of the participants (82.1%) was 19–35 years. More than 39% of the participants were unable to read and write, and 56.1% lived in urban areas (Table 1).

**Table 1:** Characteristics of participants at baseline in South East Tigray, Ethiopia, 2018 (N = 831, 731 users of injections and 100 users of pills).

Variable		Frequency	Percentage
Age	<19	49	5.9
	19–35	682	82.1
	>35	100	12
Residence	Urban	466	56.1
	Rural	365	43.9
Educational status	Unable to read and write	325	39.1
	Able to read and write	94	11.3
	Grade 1–8	192	23.1
	Grade 9–12	176	21.2
	Diploma and above	44	5.3
Ever been pregnant	Yes	758	91.2
	No	73	8.8
Marital status	Married	766	92.2
	Single	65	7.8
Occupational status	Farmer	245	29.5
	Housewife	360	43.3
	Government employee	73	8.8
	Trader	132	15.9
	Student	21	2.5

### Contraceptive characteristics

Out of 831 users, 731 (88%) used the contraceptive injection and the remaining 100 (12%) used contraceptive pills. More than half (55.4%) of those surveyed used the method to space their pregnancy timings. About 61.9% women chose the method by themselves and 88% of them obtained information about contraception from the health provider (Table 2).

**Table 2:** Characteristics of participants related to family planning in South East, Ethiopia, 2018 (N = 831, 731 users of injections and 100 users of pills).

Variable		Frequency	Percent
Types of contraceptive	Injections	731	88
	Pills	100	12
Purpose of using the method	Delay pregnancy	285	34.3
	Timing of pregnancy	460	55.4
	Prevent pregnancy	86	10.3
Who chose the method	Themselves	514	61.8
	Their partner	44	5.3
	Themselves & partner	273	32.9
	Others	514	61.8
Source of information	Health provider	731	88
	Friend	56	6.7
	Media	44	5.3
Duration of prior use of the method	<6 months	163	19.6
	6–18 months	251	30.2
	>18 months	417	50.2
Intention to continue using this method	Yes	780	93.9
	No	51	6.1
Experience of using another method	Yes	241	29
	No	590	71

### Discontinuation rate of contraceptive use

Among the 828 users at 4 months, 198 (24%) had discontinued using the method, 630 women had continued and three were lost in follow-up. Furthermore, 48 out of 100 (48%) users of pills and 150 out of 728 (20.6%) users of injections discontinued to use at four months. The most common reasons for discontinuation were side effects [84 (10.1%)] and complications [44 (5.3%)].

At 8 months, 399 out of 828 (48.2%) users discontinued to their baseline method. At this interval, 69 out of 100 (69%) pill users and 330 out of 728 (45.3%) injection users discontinued the methods. The most common reasons to discontinue at this stage were side effects (7.7%) and complications (5.7%).

At the end of the 12 months, 526 out of 828 (63.5%) users discontinued to use their baseline method. The number of pill and injection users that discontinued contraceptive use at the one year point were 84 out of 100 (84%) and 442 out of 728 (60.7%) respectively. The reasons for discontinuing at one year were side effects and complications with the same level of percentage (each 4.6%).

Out of all participants, 18 (2.17%) of them encountered contraceptive failure. Other reasons to discontinue the methods were lack of supply (59) and poor client-provider interactions (16). Discontinuation rate and reasons for discontinuation at different stages of interview are detailed in Tables 3 and 4 respectively. Users who did not use a baseline method or switched to long term methods at 4, 8 and 12 months were 163, 376 and 525 respectively.

**Table 3:** Reasons for discontinuation of contraceptive use from baseline method after 4, 8 and 12 months in South East Tigray Region, Ethiopia (N = 831, 731 users of injections and 100 users of pills).

Reasons	At 4 months %(frequency)	At 8 months %(frequency)	At 12 months %(frequency)
Side effects	42.4(84)	31.8(64)	29.9(38)
Complications	22.2(44)	23.4(47)	29.9(38)
Lack of supply	5.1(10)	11.9(24)	19.7(25)
Method failed	2.5(5)	4(8)	3.9(5)
Husband opposed	10.6(21)	10(20)	11.8(15)
Want to conceive	16.2(32)	13.9(28)	1.6(2)
Poor client and staff relationship	1(2)	5(10)	3.2(4)

**Table 4:** Discontinuation rate of family planning users in South East Tigray Region, Ethiopia, 2018 (N = 831, 731 users of injections and 100 users of pills).

Follow-up time	Types of contraception	Number of users discontinued to use baseline method	discontinuation rate
4 months	Pills	48	48
	Injections	150	20.6
8 months	Pills	69	69
	Injections	330	45.3
12 months	Pills	84	84
	Injections	442	60.7

### Switching to other methods

At the three intervals (4, 8 and 12 months), the number of users who switched to other types of family planning were 68, 51 and 31 respectively. The numbers of these who switched to implants were 28, 24 and 17 respectively. At 4, 8 and 12 months the number of users who did not use any method were 130(15.7%), 348(42%) and 495 (59.7%) respectively (Table 5).

**Table 5:** Switching characteristics of family planning users in South East Tigray Region, Ethiopia, 2018 (N = 831, 731 users of injections and 100 users of pills).

Follow up time	Number of switchers	After switch used method	Frequency (%)
4 months	68	Pills	10(14.7)
		Injections	25(36.8)
		Implants	28(41.2)
		Intrauterine contraceptive device (IUCD)	5(7.3)
8 months	51	Pills	16(31.4)
		Injections	7(13.7)
		Implants	24(47.1)
		IUCD	4(7.8)
12 months	31	Injections	1(3.2)
		Implants	17(54.8)
		IUCD	13(41.9)

### Factors associated with the discontinuation of contraception at 12 months follow-up

In order to assess the factors associated with discontinuation of contraception, the Cox proportional-hazards model was used. In the adjusted model, the following factors showed a significant association with discontinuation of contraceptive use; provision of family planning services with another maternal health clinic, the profession of the provider, the type of contraception used and the intention to continue to use the baseline method.

The use of pills was associated with discontinuation within one year (HR = 1.77; 95% CI = [1.4–2.3]). Women who were used pills were 1.77 times more likely to discontinue. The delivery of family planning services in the same room as other services (i.e. no dedicated, private space for family planning services) was 1.58 times more likely to discontinuation (HR = 1.58; 95% CI = [1.16–2.2]). Similarly, receiving family planning services from a health officer was significantly associated with discontinuation (HR 3.7[95% CI 1.66–8.2]). Users received services from health officer were 3.7 times risk of discontinue as compared with users received service from health extension workers. Not intending to use the baseline method continuously was also associated with discontinuation (HR = 1.6; 95% CI = [1.16–2.24]). Users not intending to use the baseline method were 1.6 times more likely to discontinue as compared with users intending to use the baseline method (Table 6).

**Table 6:** Hazard ratio for the risk of discontinuation at 12 months among family planning users in South East Tigray Region, Ethiopia, 2018 (N = 831, 731 users of injections and 100 users of pills).

Variable		Crude model		Adjusted model	
		HR	95%CI	HR	95%CI
Age	<19	1.6	1.06–2.5		
	19–35	1.3	0.99–1.77		
	>35	Ref			
Have separate room for family planning service	Yes	Ref			
	No	1.68	1.24–2.27	1.58	1.16–2.2
Profession of the provider	Health extension worker	Ref			
	Midwife	1.5	0.8–2.7	1.6	0.87–2.9
	Nurse	1.59	0.8–2.9	1.6	0.88–3
	Health officer	3.47	1.59–7.58	3.7	1.66–8.2
Contraception types	Pill	1.8	1.4–2.3	1.77	1.4–2.3
	Injection	Ref			
Intention continue to use the baseline method	Yes	Ref			
	No	1.76	1.28–2.4	1.6	1.16–2.24
Informed of next appointment	Yes	Ref			
	No	1.5	1.02–2.3		
Duration using the current method	< = 24 month	1.3	1.1–1.6		
	>24 months	Ref			

**Fig 2:**
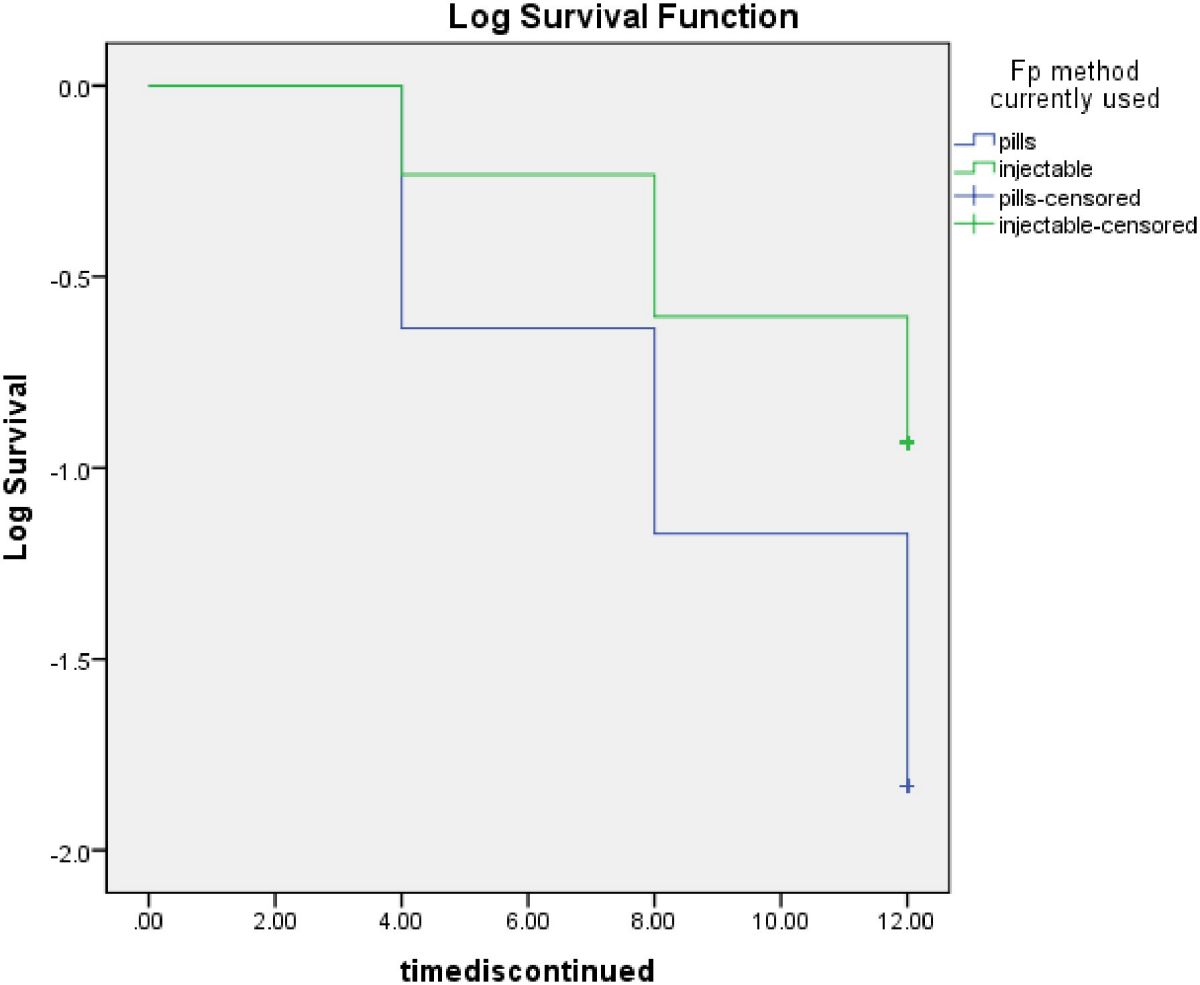
Kaplan-Meier survival curve for 12-month continuation of short acting contraceptive: Pill and injection users, Breslow chi-square p <0.001.

Continuation of contraceptive use was stratified by method (pill and injection). At 12 months, there was a significant difference in the discontinuation rate of those using pills compared to injections, where the performance of the latter is better. The Breslow-Wilcoxon test equality survival distributions for pills and injections (p = <0.001) are shown in Fig 2.

## Discussion

The objective was to assess the discontinuation rate of the contraceptive pill and injection using prospective follow-up study. Our findings showed that around two thirds of users discontinued using pills and injections within 12 months. Discontinuation of using pills and injections varied with other factors, namely the presence of other services in the same room as family planning, the intention of the woman to use the method continually, the type of method used and the profession of the person providing the method. The rate of discontinuation among users of pill and injections in our study was high when compared to two studies conducted in the USA that found discontinuation rates of 43% for injections, but 45% for pills [11], and 42.9% for injections compared to 39.4% for pills [12]. This could be due to the study setting, in USA the awareness of users might be better than from these participants in Ethiopia. A study conducted in Urban Senegal found discontinuation rates at 12 months to be 38% for pills and 32.7% for injections [13]. The reason for higher figures in other studies may be related to the study area, all the study participants were from the urban areas and this could have contributed to the low discontinuation rate as compared to this study setting. Users from urban have high exposure to different contraceptive methods and the same have high tolerance of method related confusions.

Other studies have reported discontinuation rates from Iran (55.4%) [10], Democratic Republic of Congo (22%) [14], East Azerbaijan (57.5%) [15] and urban Ghana (29%) [16] to be lower in magnitude than found in this study. Similarly, research in urban Honduras (45%) [5], Bolivia (49%) [17] and St. Louis, USA (51.5%) [12] has revealed lower discontinuation rates than our study. Conversely, the present finding was lower in comparison to that reported in California, USA (81.5%) [9] and Rocky Mountains, USA (77%) [18]. Furthermore, higher discontinuation rate figures for pills (70%) and injections (38%) are reported in the 2011 EDHS [2]. These variations could be related to differences in the study area. The implication is that studying the continuation rate of contraceptive use can be challenging and due attention should be given to improve continuity of contraceptive use.

The majority of the participants chose injections rather than pills (87.9% Vs 12.07%). This finding is supported by the 2019 mini EDHS where injection was the most popular method used, and pills much less popular (27% Vs 2%) [3]. This difference may be attributed to users promoting their method of choice, the attitude of the family planning provider, and the prevalence and severity of side effects.

The main reason for discontinuation was the side effects of the contraceptive method. Findings from Rocky Mountains [18], Democratic Republic of the Congo [14], Iran [10], Senegal [19] and the 2016 EDHS [20] support this assertion, since side effects were the main reasons for the termination of the baseline method. A report on expanding contraceptive choice found side effects to be the main reason for discontinuation [4]. This is might be due to the family planning provider not adequately counseling the user on the possible side effects of the chosen method and how to handle them. I.e. if the users are not aware on possible tolerable side effects, they may consider it as abnormality or complication related to the method and finally they will decide to terminate it. So proper counseling will create awareness on the types of side effect, when could be tolerated, and when it will need special attention.

We found higher discontinuation rate for pill users as compared to injection users. A similar outcome was also presented in a study in Benin [21]. The reason for the disparity might be related to the challenges in deciding and remembering to take the pill daily, with regard to any side effects the user may be experiencing, whereas contraceptive injections are taken every 3 months and their side effects cannot be altered subsequently.

In our study, not having the intent to use the method for one year was significantly associated with discontinuation. This is consistent with research in California, USA [9]. The finding is also supported by the theory of reason—if a person plans to do something, then there is a high likelihood of them doing it [9].

The discontinuation rate for participants receiving family planning services with other services (antenatal and postnatal clinics) was higher compared to users receiving their service in a separate room. A study in Banyuning Village (Indonesia) also showed that the quality of service can affect the continuous use of contraception [22]. Hence, quality of service may have a great impact in the continuation rate of contraceptive use. One way of improving the quality of service is to maintain the privacy of the user. If the facility provides multiple services (antenatal and postnatal clinics) in the same area, the healthcare providers may not have enough time to thoroughly counsel the woman about the characteristics of each contraceptive method.

Another factor to discontinuation was the contraceptive service being provided by a health officer as opposed to midwives or nurses. This might be due to the better counseling skills of midwives and nurses due to the content of training they receive, which is highly focused on counseling, also on the post-graduate work experience midwifes and nurses receive by working in maternal health clinics including in family planning.

### Limitation of this study

it is only focused on short acting family planning pills and injection, so we recommended other researchers to conduct by considering the other short acting and long acting family planning methods.

## Conclusion and recommendation

The one-year discontinuation rate of pills and injections was high. To decrease the discontinuation rate of contraception, health facilities should set aside separate rooms for family planning services, and the service should be provided by midwives and nurses who have good skills in counseling. To decrease discontinuation due to side effects, it is possible to provide reassurance to the user to maintain continuous use of the method as most side effects are manageable.

## Supporting information

S1 Data Short acting SPSS main. (SAV)
